# Effect of spray air settings of speed-increasing contra-angle handpieces on intrapulpal temperatures, drilling times, and coolant spray pattern

**DOI:** 10.1007/s00784-021-04030-3

**Published:** 2021-06-18

**Authors:** Edina Lempel, József Szalma

**Affiliations:** 1 Department of Conservative Dentistry and Periodontology, Medical School, University of Pécs, 5. Dischka St, 7621 Pécs, Hungary; 2 Department of Oral and Maxillofacial Surgery, Medical School, University of Pécs, 5. Dischka Gy St, Pécs, 7621 Hungary

**Keywords:** Aerosol, SARS-CoV-2, iInfection control, High-speed drilling, Pulp temperatures

## Abstract

**Objectives:**

Decreasing aerosol leaks are of great interest, especially in the recent era of COVID-19. The aim was to investigate intrapulpal heat development, coolant spray patterns, and the preparation efficiency of speed-increasing contra-angle handpieces with the spray air on (mist) or off (water jet) settings during restorative cavity preparations.

**Methods:**

Standard-sized cavities were prepared in 80 extracted intact human molar teeth using diamond cylindrical drills with a 1:5 speed-increasing contra-angle handpiece. A custom-made device maintained the standardized lateral drilling force (3 N) and predetermined depth. Temperatures were measured using intrapulpal thermocouple probes. The four experimental groups were as follows: mist cooling mode at 15 mL/min (AIR15), water jet cooling mode at 15 mL/min (JET15), mist cooling mode at 30 mL/min (AIR30), and water jet cooling mode at 30 mL/min (JET30). The coolant spray pattern was captured using macro-photo imaging.

**Results:**

The JET15 group had the highest increase in temperature (ΔT = 6.02 °C), while JET30 (ΔT = 2.24 °C; *p* < 0.001), AIR15 (ΔT = 3.34 °C; *p* = 0.042), and AIR30 (ΔT = 2.95 °C; *p* = 0.003) had significantly lower increases in temperature. Fine mist aerosol was formed in the AIR15 and AIR30 preparations but not in the JET15 and JET30 preparations (*p* < 0.001). The irrigation mode had no influence on the preparation time (*p* = 0.672).

**Conclusions:**

Water jet irrigation using coolant at 30 mL/min appeared to be the optimal mode. Considering the safe intrapulpal temperatures and the absence of fine mist aerosols, this mode can be recommended for restorative cavity preparations.

**Clinical significance:**

To increase infection control in dental practices, the water jet irrigation mode of speed-increasing handpieces with coolant flow rates of 30 mL/min should be considered for restorative cavity preparations.

**Supplementary Information:**

The online version contains supplementary material available at 10.1007/s00784-021-04030-3.

## Introduction

Tooth enamel and dentin preparations—as hardest materials in the human body—may have unfortunately unpleasant temperature consequences [1]. The specifications of the drill (drill material and geometry) and drilling conditions (revolution speed, axial pressure, and irrigation method) strongly influence intrapulpal temperatures intraoperatively [2]. For restorative and prosthetic tooth preparations, air-turbines and high-torque electric handpieces are frequently used, which require constant water coolant due to heat generation [3]. These high-speed rotating instruments generate spray, resulting from a mixture of the water coolant and “chip air” [4].

Dental aerosols (particles < 10 μm) and splatter (particles ≤ 50 μm) often include saliva and/or blood [5, 6]. Furthermore, the saliva of individuals infected with severe acute respiratory syndrome coronavirus 2 (SARS-CoV-2) contains this virus, regardless of the presence of symptoms [7–10]. Infected patients may have 9.9 × 10^2^–1.2 × 10^8^ viral copies per mL of saliva [7, 11]. Furthermore, salivary viral load can be detected up to 25 days after the onset of symptoms [7, 11].

There are two main mechanisms by which aerosol are generated when using high-speed handpieces [4]. One mechanism is the pre-misted and premixed water coolant and “chip air” and the second is the atomization effect of the high-speed rotating bur ejecting high-speed droplets into the environment [4]. When an air turbine is used, contaminated aerosol particles can be found 4 m from the dental unit, and the fine mist spray particles can be projected at speeds that exceed 12 m/s [4, 5].

Potential methods of managing exposure to aerosols include applying more effective high-volume evacuators, using individual protective transparent acrylic chambers, increasing mechanical ventilation using air cleaners and filters, and reducing the formation of aerosol spray by decreasing either the rotational speed of the drills or the amount of water coolant [3, 6, 12–18]. However, reducing irrigation may result in more limited visualization during fine and precise movements; moreover, chip clogging of the drill and increased friction can occur [6, 19]. With a less effective drill, preparation times and temperatures may also increase [1, 20]. Higher and longer lasting temperature peaks, and specifically those exceeding the 5.5 °C increase threshold, may lead to pulpal necrosis, and an excessive temperature increase of 3–10 °C can lead to periodontal malformations (e.g., alveolar bone necrosis, bone loss, and ankylosis) [1, 21]. Based on previous research, a flow rate of 30–50 mL/min of coolant is adequate [22–24], although the International Organization for Standardization recommended a rate of 50 mL/min in 2017 [25]. In some recently developed speed-increasing contra-angle handpieces, the “chip air” can be switched off to avoid pre-misting the coolant in the instrument head. For other manufacturers, the spray air tube connecting the hose and the treatment center can be blocked by a pin produced by the manufacturer. Although blocking spray air has recently been developed to reduce aerosol dispersion, to the best of our knowledge, its effect on restorative cavity preparations or on intrapulpal temperatures has not yet been evaluated.

Therefore, the aim of this study was to investigate the increase in intrapulpal temperature, preparation efficiency, and the coolant patterns of speed-increasing contra-angle handpieces using the spray air on (coolant mist) and spray air off (water jet coolant) modes during restorative cavity preparations. The tested null hypothesis of this study was that spray settings and reduction in coolant volume has no significant effect on preparation circumstances regarding pulpal heat, fine mist aerosol formation, and preparation times.

## Materials and methods

All the used materials and equipment of this study were collected in Table 1.

**Table 1:** The used materials and equipment in this study

Name of the material/equipment	Manufacturer
Iwanson-caliper	Hager & Werken GmbH & Co., Duisburg, Germany
Thermocouple probe (*T-type Cu/CuNi*)	TC Direct, Budapest, Hungary
Flow composite (*Filtek Supreme Flowable Restorative*)	3 M, St. Paul, MN, USA
Thermal paste (*Arctic Silver 5*)	Scan Computers International Ltd., Bolton, UK
Dental adhesive (*Adper Single Bond 2*)	3 M, St. Paul, MN, USA
Digital thermometer (*EL-EnviroPad-TC*)	Lascar Electronics Ltd., Salisbury, UK
Non-contact thermometer (*Testo845*)	Testo Magyarország Kft., Budapest, Hungary
1:5 speed-increasing contra-angle handpiece (*TiMax Z95L*)	NSK-Nakanishi, Eschborn, Germany
Medium-grit diamond cylindrical drill (*No. 837*)	Hager & Meisinger, Neuss, Germany
Dental unit (*KaVo Esthetica E30S*)	Kaltenbach & Voigt GmbH, Biberach, Germany
Digital jewelry scale (*SBS-LW-500*)	Steinberg Systems, Berlin, Germany
Full-frame camera (*Canon EOS RP*)	Canon, Huntington, USA
Prime lens (*EF 100 mm f/2.8 L Macro IS USM*)	Canon, Huntington, USA
Studio flash (*Godox MS 300*)	Godox Photo Equipment Co., Ltd., Shenzhen, China
Softboxes (*Godox 90* × *60*)	Godox Photo Equipment Co., Ltd., Shenzhen, China
Light/lux meter (*Voltcraft LX-10*)	Conrad, Budapest, Hungary
Laptop computer (*X1 Carbon sixth gen.*)	Lenovo, Beijing, China

### Preparation of tooth specimens

**Fig. 1:**
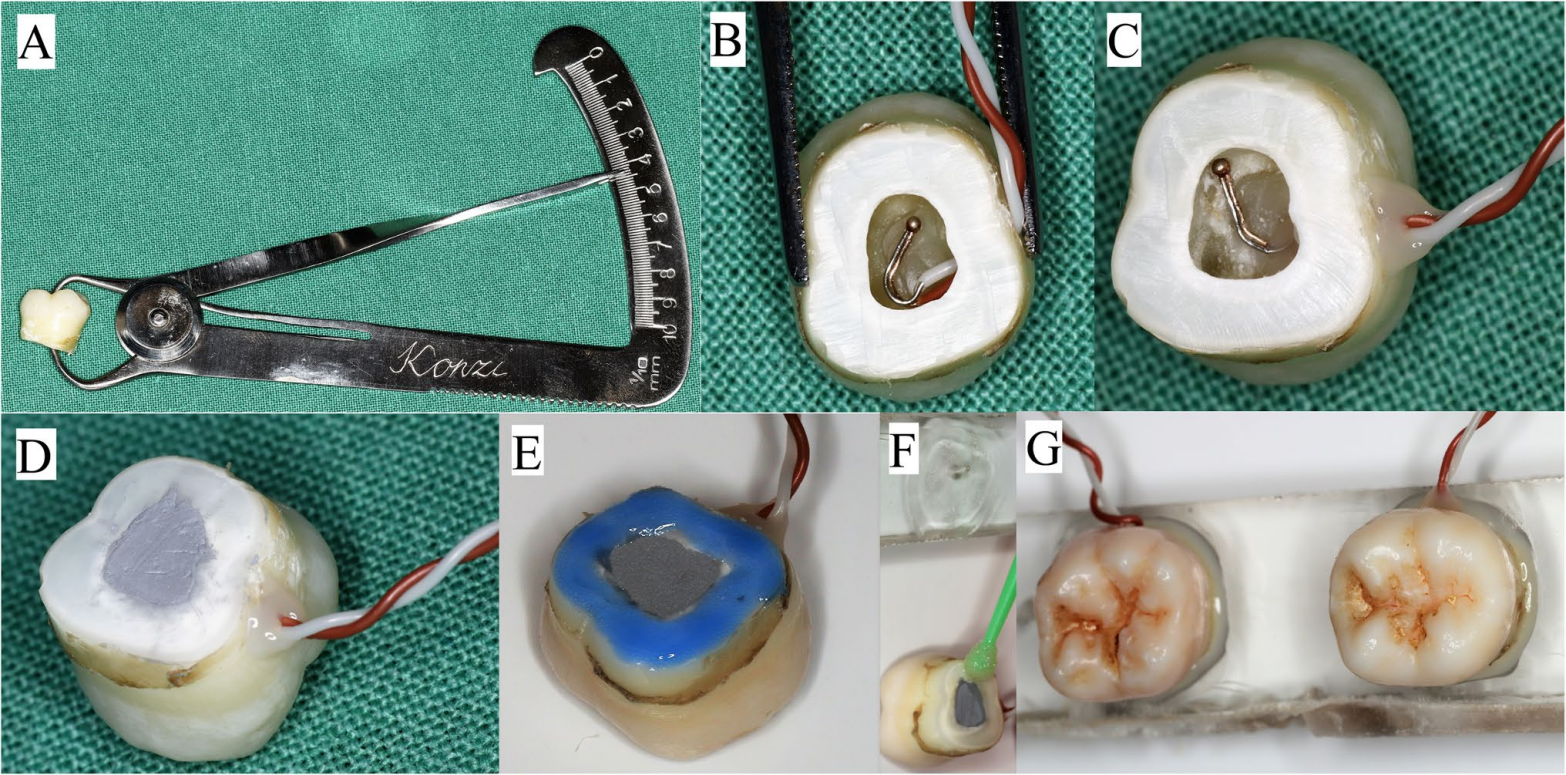
Tooth specimen preparation **A** distance between the pulp chamber roof and the central fissure was measured using an Iwanson-caliper; **B** the thermocouple probe was introduced into the pulp chamber through a small buccally/lingually prepared hole; **C** the probe was fixed and the penetration hole was closed with flow composite material; **D** the pulp chamber was filled with thermal paste; and **E**, **F**, **G** after etching and bonding, the tooth was fixed on an acrylic plate

In this in vitro study, 80 freshly extracted intact human molar teeth were used (Regional Research Ethical Committee approval: No. PTE/3795). The teeth used were extracted because of periodontitis or they were intact third molars removed for pathologic or orthodontic indications. Crowns selected for the study were free from any drilling injuries or caries. After extraction, the teeth were stored in 4% paraformaldehyde. Teeth were sectioned 3 mm below the cemento-enamel junction line (coronectomy). The pulp tissue was extirpated completely from the pulp cavity using excavators. An Iwanson-caliper (Hager & Werken GmbH & Co., Duisburg, Germany) was used to measure and register the distance between the top of the pulp chamber and the central fissure (Fig. 1). The exact drilling depth was calculated to leave a 1-mm thick intact dentin surface between the prepared cavity and the pulp chamber. When irregular mesial and distal pulp horns created an uneven, inclined restorative cavity base, the tooth was placed on the drilling tower (described below in “Experimental setup”) with respect to the required inclination.

### Temperature measurements

**Fig. 2:**
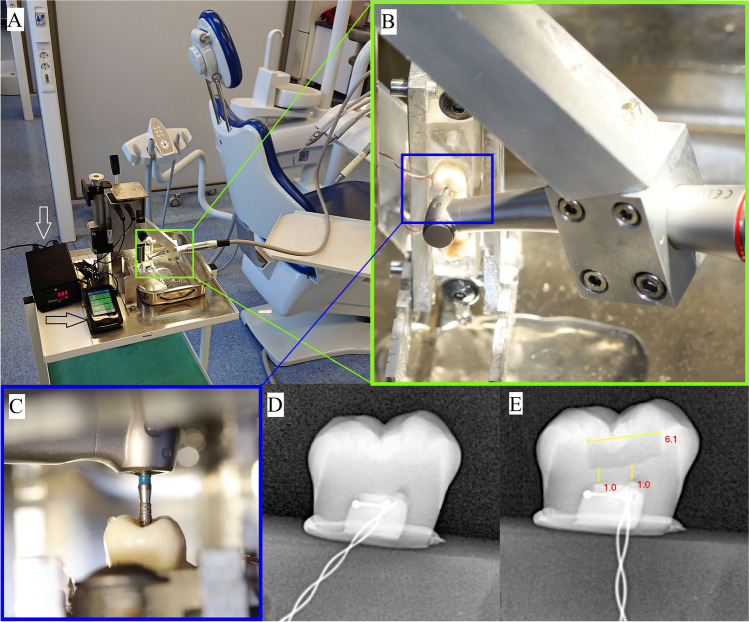
The drilling tower and restorative cavity drilling **A** the drilling tower was attached to a regular dental clinical unit. The time measurement unit (white arrow) and temperature registration device (black arrow) cooperated with the drilling tower; **B** two molars were attached to each acrylic plate; **C** the drill’s initial position in the hole in the tooth (as the starting point of cavity preparation); **D** before drillings, the placement and position of the thermosensor was controlled with intraoral radiographs; **E** after cavity preparations, the preoperative caliper measurement method was regularly controlled. It is clear that drilling with inclination was possible with the adjustment of the acrylic plate holding the metal frame in the tower

A 0.5 mm diameter T-type Cu/CuNi thermocouple probe (TC Direct, Budapest, Hungary) was attached to the roof of the pulp chamber and fixed with a small drop of flow composite (Filtek Supreme Flowable Restorative, 3 M, St. Paul, MN, USA) (Fig. 1). The probe’s detecting bimetal wire was placed mesiodistally for the full length of the pulp chamber. The chamber was filled with thermal paste (Arctic Silver 5, Scan Computers International Ltd., Bolton, UK), and the tooth was glued with dental adhesive (Adper Single Bond 2, 3 M, St. Paul, MN, USA) on acrylate plates hermetically closing the caudal surface. Thermocouple sensors were coupled with a digital thermometer (EL-EnviroPad-TC, Lascar Electronics Ltd., Salisbury, UK) at a resolution of 0.1/1 °C and a data sampling frequency of 1 measurement per second (Fig. 2).

### Experimental setup

Experiments were conducted in a dental clinical teaching unit at the University of Pécs, Medical School, Department of Dentistry, Oral and Maxillofacial Surgery (Pécs, Hungary). During the experiment, the room temperature was maintained at 27 ± 0.5 °C with air-conditioning devices. The coolant temperature of the water reservoir of the dental unit was maintained at 26 ± 0.5 °C, which was controlled before each drilling and corrected if necessary. Room temperature and water temperature were measured using a precision non-contact thermometer (Testo845, Testo Magyarország Kft., Budapest, Hungary).

For the experiment, a 1:5 speed-increasing contra-angle handpiece (TiMax Z95L, NSK-Nakanishi, Eschborn, Germany) was used with a medium-grit diamond cylindrical drill (Ø = 1.4 mm, length of diamond coating = 6 mm) (837, Hager & Meisinger, Neuss, Germany). The connected dental unit (KaVo Esthetica E30S, Kaltenbach & Voigt GmbH, Biberach, Germany) maintained a constant revolution speed (40,000 revolutions per minute [rpm]) and a constant irrigation flow rate. The irrigation flow rate was adjusted beforehand using a measuring glass and a stopwatch and set to 15 mL/min or 30 mL/min, depending on the experimental group.

The rotational speed of the drill was 200,000 rpm (40,000 rpm of the dental unit × 1:5 acceleration of the handpiece). The calculated cutting speed [= *(*diameter in meter × rpm × π)/60] of this drill during the experiment was 14.65 m/s. One drill was used to prepare five cavities and then was exchanged for a new drill.

**Fig. 3:**
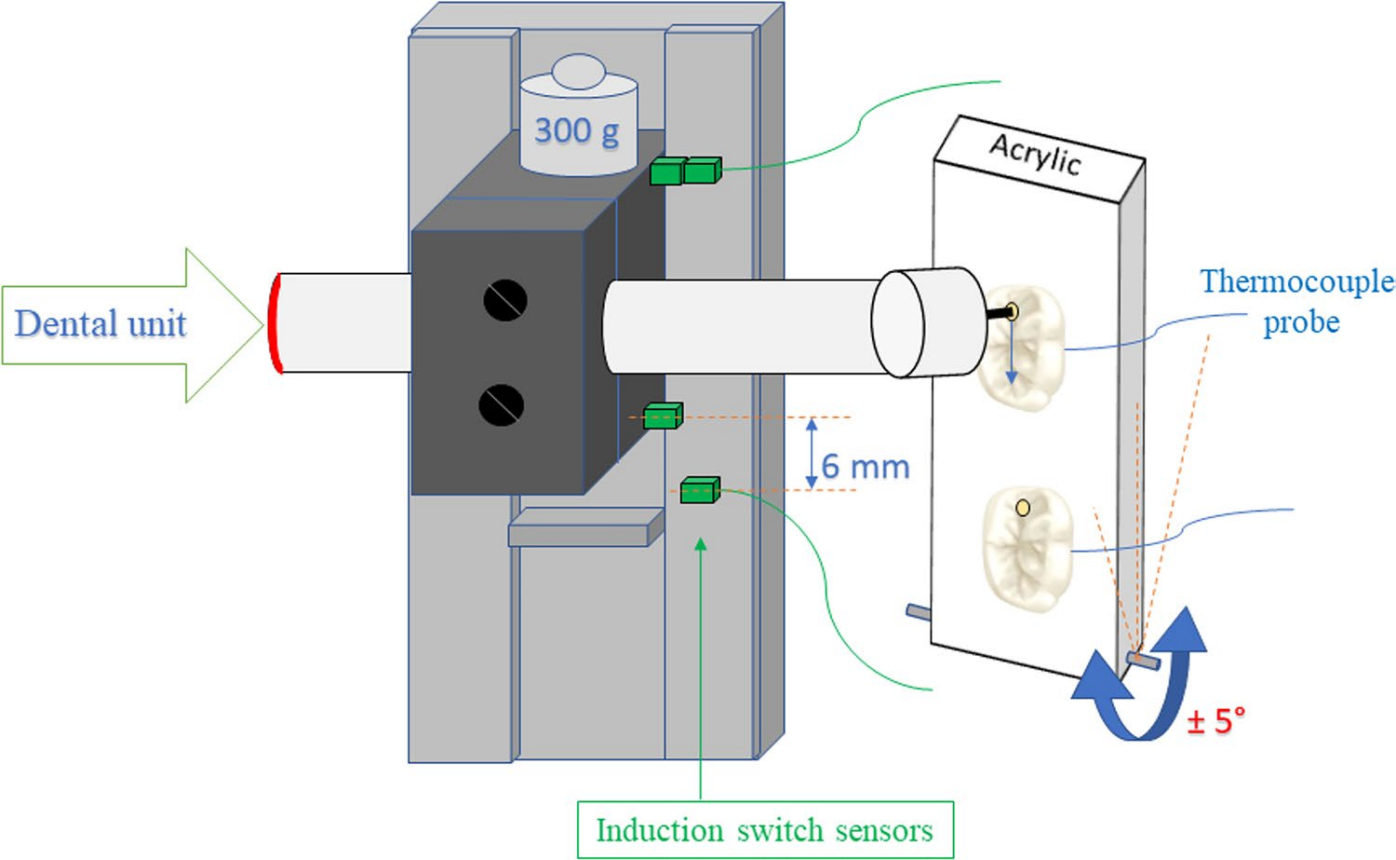
The simplified schematic illustration of the experimental setup. The standard lateral drilling force was maintained with the weight of the moving parts (including the handpiece) of the tower. The axial range of the moving part was set to 6 mm (identical to the cavity length), while the drilling times required for this 6-mm axial movement were measured automatically with the help of built-in induction sensors and an electrical switch unit

For the experiment, a custom-made drilling tower was used to fix the speed-increasing contra-angle handpiece and maintain the axial load (lateral drilling force) and drilling length constant (Figs. 2 and 3). Before securing the acrylate plate with the crowns into the drilling tower, a 1.4 mm in diameter initial hole preparation was necessary in the mesial pit of the fissures to allow the drills to be fixed later in the correct starting position in the tower device before the standard experimental cavity preparations. The depth of the initial hole was dependent on the distance between the central fissure and the roof of the pulp chamber (3.5–4.5 mm). The vertically oriented acrylate plate securing the sectioned crown on its lateral side was fixed to a metal frame attached to the determined slot of the drilling tower. The vertical orientation of the occlusal surface allowed a downward movement of the horizontally orientated drill along the central fissure (Fig. 3). The horizontally orientated handpiece with the drill moved only in the vertical plane in-line with the central fissure, which was maintained by the tower’s moving part. The vertical movement (i.e., drilling length) of the tower was set to 6 mm. Time measurement started and stopped automatically, because at the endpoints of this 6 mm vertical movement section, magnetic induction switches were built-in and cooperated with the time measurement unit.

In cases where unequal pulp horns caused the cavity floor to be inclined, the angle of the tooth in the tower device was modified by adjusting the vertical axis of the metal fixating frame holding the acrylic plate. Additional fine, minor adjustments were possible also during drilling.

The pressure force was determined and maintained at 3 N (300 g) during drilling [19, 26, 27]. The combined weight of the handpiece and the tower’s moving part was completed with metal weights to get the required 300 g overall axial load. The weight appearing at the tip of the drill was controlled with a digital jewelry scale (SBS-LW-500, Steinberg Systems, Berlin, Germany). Before drilling, the metal frame and acrylic plate holding and positioning the teeth were stored in physiological saline solution at 37 °C in a thermostat-controlled sink bath. These parts were removed from the bath only right before starting the drilling.

**Fig. 4:**
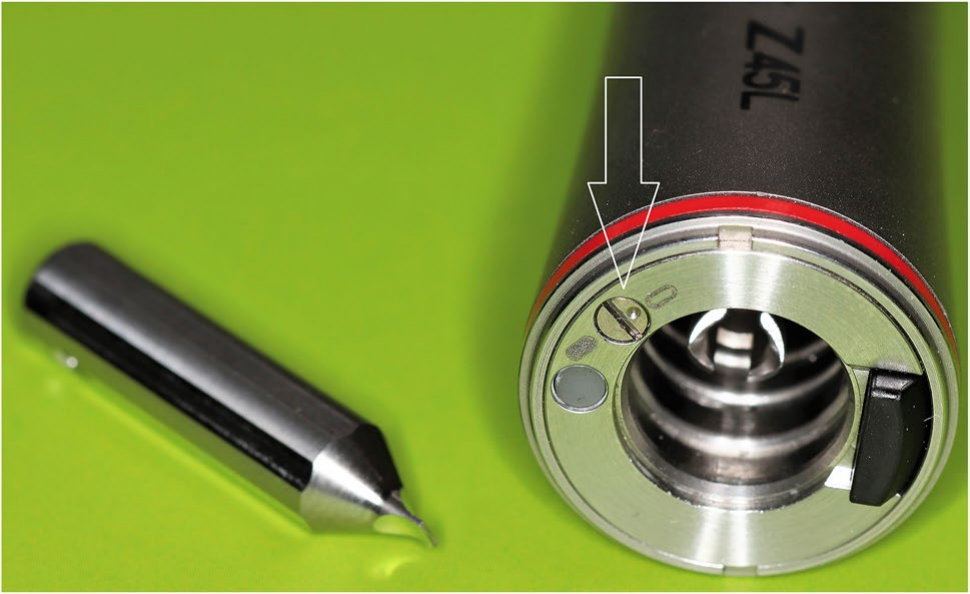
The investigated speed-increasing contra-angle handpiece gave the possibility to switch easily between “spray air on” and “water jet” irrigation modes with the tiny screw (white arrow) and the fitting screwdriver

The handpiece had a patented spray switch function. To switch between the irrigation modes (water jet vs. mist mode), a screw at the base of the handpiece had to be turned 180° clockwise or anticlockwise with a screwdriver that was provided by the manufacturer (Fig. 4).

In the first and third experimental group (*n* = 20 teeth each), the spray air was switched on (i.e., mist mode) and the irrigation flow rate was set to 15 mL/min and 30 mL/min (AIR15 and AIR30, respectively). In the second and fourth group (*n* = 20 teeth each), the spray air of the handpiece was switched off (i.e., water jet cooling mode) and the flow rate was set to 15 mL/min and 30 mL/min (JET15 and JET30, respectively).

### Pattern of coolant spray and fine mist aerosol visualization

The pattern of the coolant spray in the different coolant modes was macro-photographed using standard setting parameters (see “Results,” Figs. 7 and 8). Full-frame cameras with a prime lens and a studio flash with 90 × 60 cm softboxes were used (Canon EOS RP camera body; EF 100 mm f/2.8 L Macro IS USM Lens, Canon, Huntington, USA and Godox MS 300, GODOX Photo Equipment Co., Ltd., Shenzhen, China). The aerosol spray was captured in two positions: one with contact between the drill and tooth cavity and the other without contact.

For image analysis, Windows Photo Viewer (Microsoft Corp., Redmond, USA) software was used on a laptop computer with full HD screen resolution (1920 × 1080 pixels) (X1 Carbon sixth gen., Lenovo, Beijing, China). The ambient light was reduced to less than 50 lx during the observations (lux meter, Voltcraft LX-10, Conrad, Budapest, Hungary). Brightness (+ 70%) and contrast (− 30%) of the images were adjusted for visualization purposes. The black background allowed displaying of single, isolated larger drops or mist-like aerosol formed during the operation of the handpiece. The coolant pattern was classified based on whether isolated water droplets consisting of one or more projectiles were clearly visible without magnification of the images or whether a homogenous mist consisting of uncountable fine and ultrafine inseparable particles were visible even in the maximally magnified images in the software. Both authors examined 20 images per group (10 with tooth cavity contact and 10 without) and confirmed either clear visibility or absence of water drops or fine mist. The pictures were saved in four different folders on the camera’s SD card. To organize the images and reduce observer bias, a technician who was not involved in the study renamed and saved each file with an 8-digit code. A total of 160 decisions were registered (4 study groups × 20 images × 2 observers). Intra- and inter-observer reliability was tested using kappa statistics.

### Statistical analysis

Data collection and statistical analyses were performed using SPSS® version 27.0 (SPSS, Chicago, IL, USA). The Kolmogorov–Smirnov test was used to test the normality of the distribution of the data. Based on the results of the Kolmogorov–Smirnov test (*p* < 0.05) non-parametric test was applied. The differences in heat production in the pulp chambers and the differences in preparation times between the investigated groups were tested using the non-parametric Kruskal–Wallis test followed by Dunn’s pairwise comparisons. The differences in the occurrence of fine mist in the AIR vs. JET groups with identical coolant flow rates were tested using Fisher exact test. *P* values below 0.05 were considered significant. Cohen’s kappa statistic was used to calculate agreement between the observers. A kappa value less than 0.40 was considered poor agreement; a value between 0.40 and 0.59 was considered moderate agreement; a value between 0.60 and 0.74 was classified as good agreement, and a value between 0.75 and 1.00 was classified as excellent agreement.

## Results

**Fig. 5:**
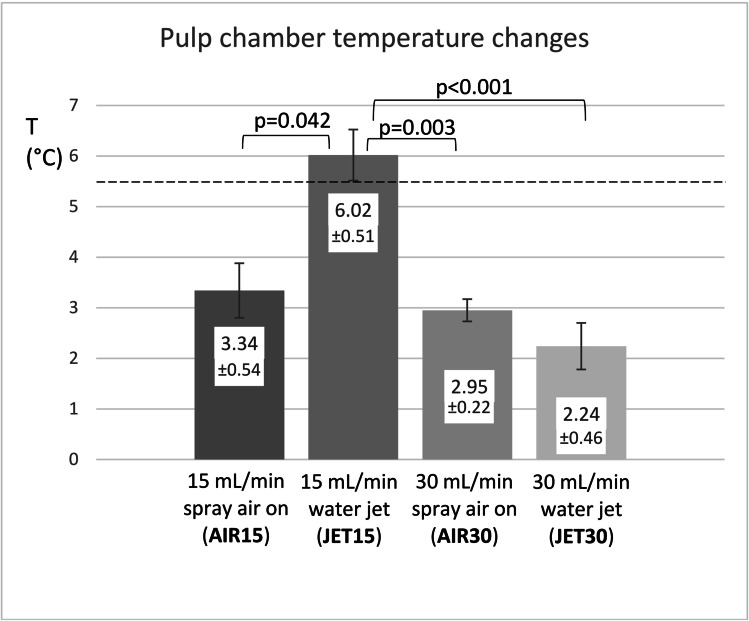
The mean increases and standard deviations in temperature measured in the pulp chambers according to the different irrigation modalities during the restorative cavity preparations. The dashed line indicates the pulp damage threshold. The Kruskal–Wallis test was performed followed by Dunn’s pairwise comparisons

The average increase in temperature in the pulp chambers did not differ significantly between the AIR15, AIR30, and JET30 preparations. The JET15 group, however, produced the highest increases in temperature in the pulp chamber (ΔT = 6.02 ± 0.50 °C). The increase in temperature was significantly lower in the JET30 (ΔT = 2.24 ± 0.46 °C; *p* < 0.001), AIR15 (ΔT = 3.34 ± 0.54 °C; *p* = 0.042), and AIR30 (ΔT = 2.95 ± 0.22 °C; *p* = 0.003) preparations (Fig. 5).

**Fig. 6:**
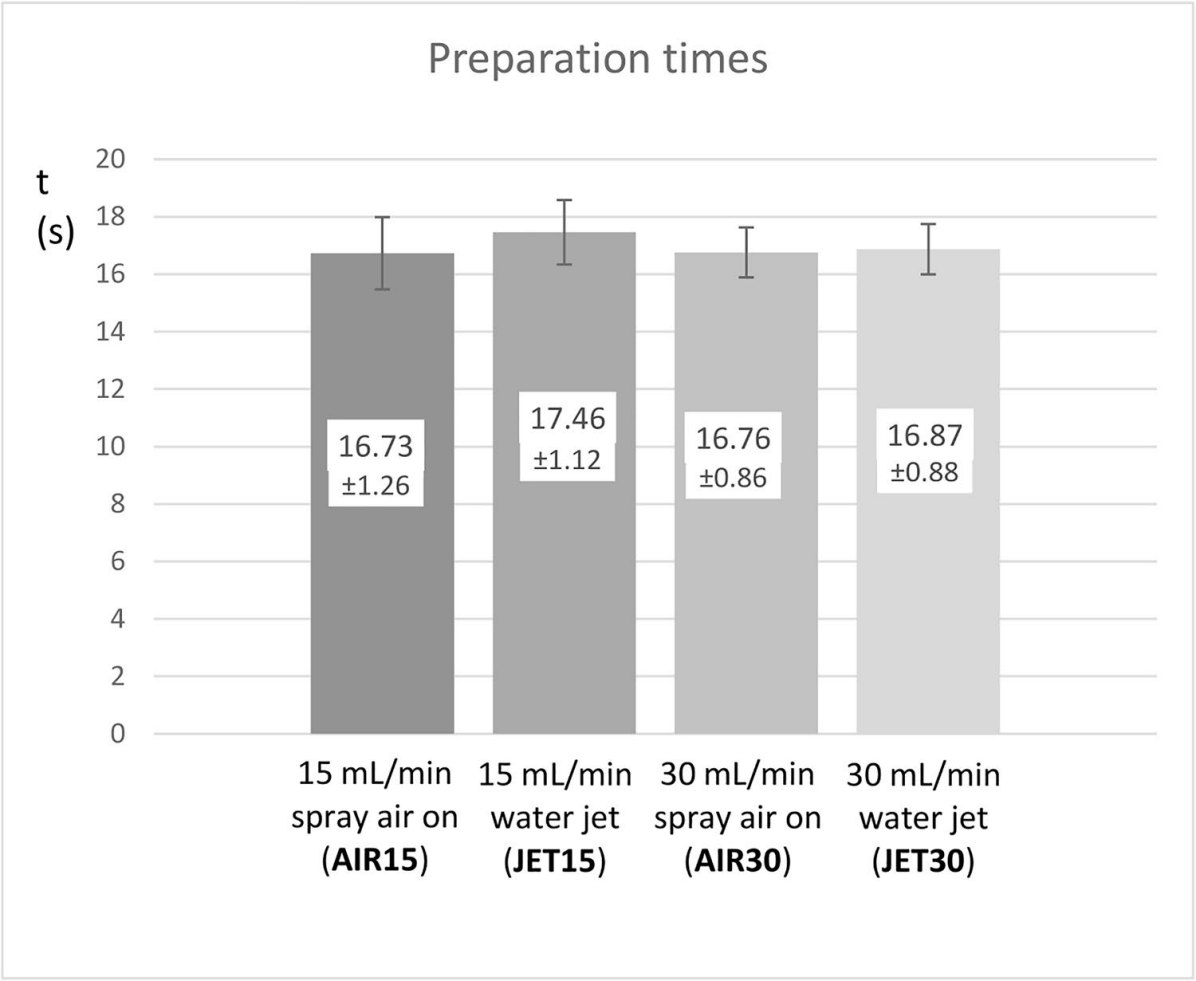
The mean drilling times and standard deviations according to the different irrigation modalities during restorative cavity preparations. Kruskal–Wallis test (*p* = 0.672)

On average, 16.7–17.5 s (min: 14.6 s; max: 19.5 s) were necessary to prepare the standard 6 mm cavities, and no statistical difference was found between the study groups (*p* = 0.672; Kruskal–Wallis test) (Fig. 6).

**Fig. 7:**
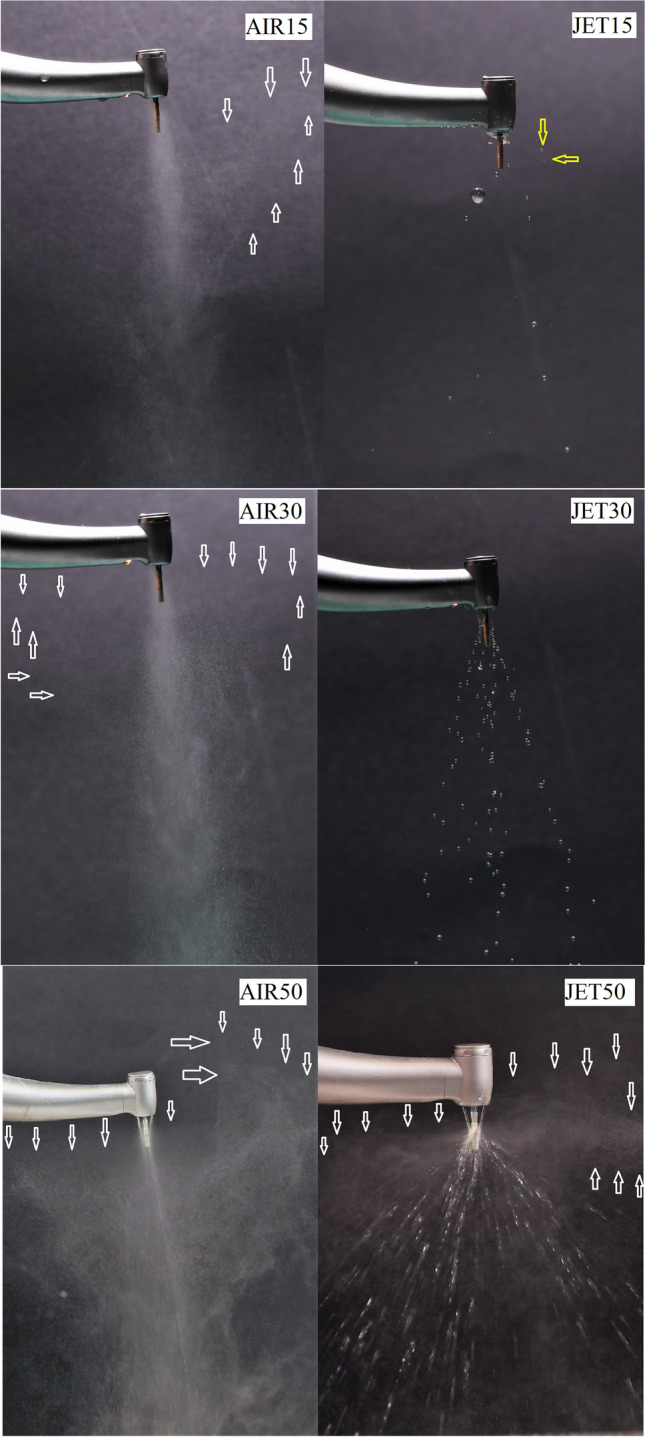
The coolant spray pattern of the speed-increasing contra-angle handpiece in the different irrigation modes. Revolutions were maintained at 200,000 rpm. AIR: “spray air on” (mist irrigation mode). JET: “spray air off’ (water jet irrigation mode). 15/30/50^*^: the coolant flow rate in mL/min. *AIR15*: fine mist aerosol formation also in radial direction is visible (white arrow). *JET15*: fine mist aerosol formation is absent, random, and radial water droplet projectiles are visible (yellow arrows). *AIR30*: fine mist aerosol formation also in radial direction is visible (white arrow). *JET30*: fine mist aerosol formation is absent; water droplets are well-structured and organized, radial projectiles are lacking. *AIR50*: Radial fine mist aerosol formation is pronounced. *JET50*: pronounced water jet is visible, but radial fine mist aerosol formation also appears due to radial atomization effect (white arrows). A single asterisk (^*^) indicates 50 mL/min drillings were illustrated only for comparisons

**Fig. 8:**
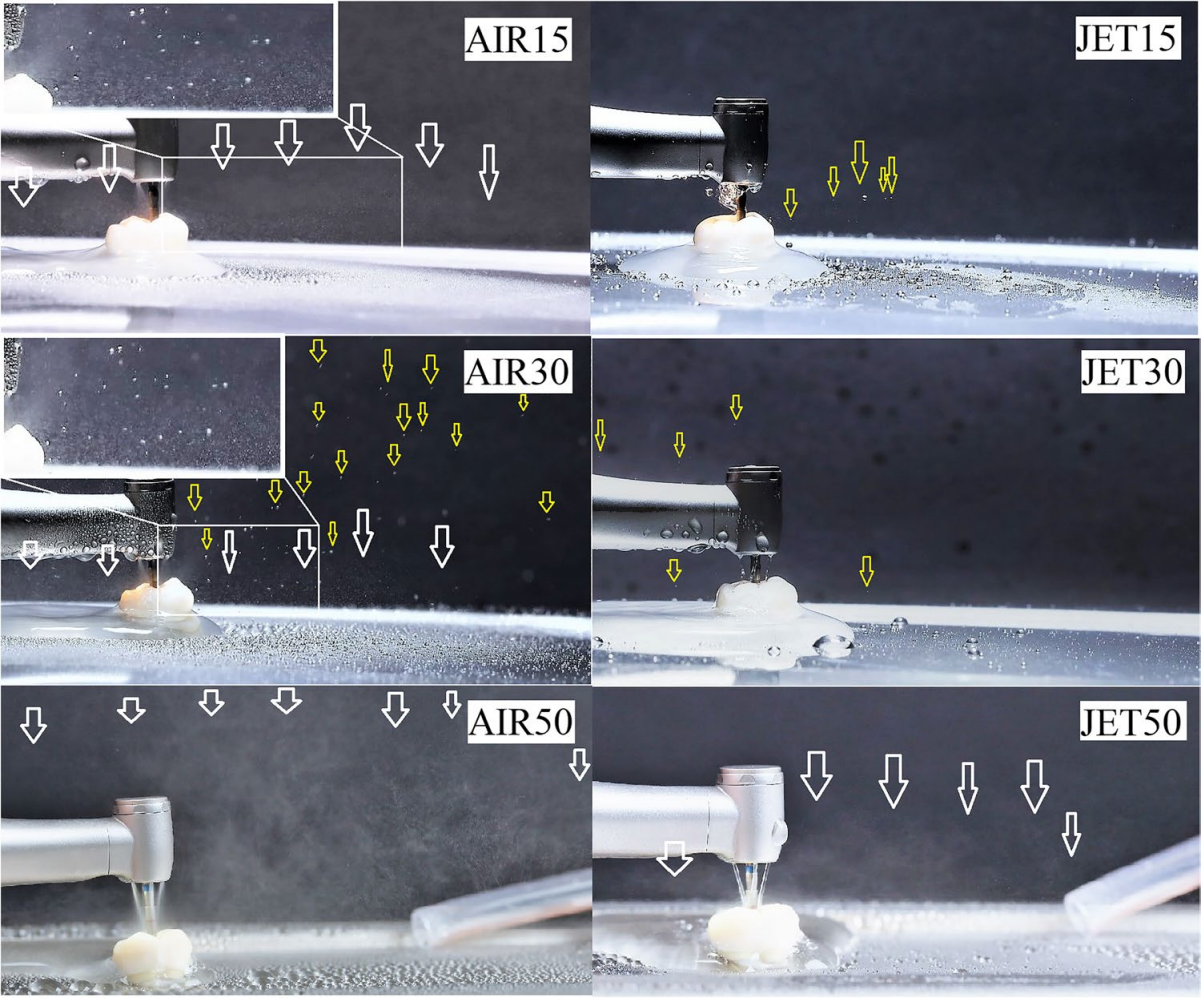
The coolant spray pattern of the different irrigation modes during restorative cavity drilling. Revolutions were maintained at 200,000 rpm. AIR: “spray air on” (mist irrigation mode). JET: “spray air off” (water jet irrigation mode). 15/30/50^*^: the coolant flow rate in mL/min. *AIR15*: fine mist aerosol formation is visible (white arrows and magnified area). *JET15*: fine mist aerosol formation is absent, random, and irregular water droplet projectiles are visible (yellow arrows). *AIR30*: radial fine mist aerosol formation is visible (white arrows and magnified area) and large amount of random water droplets are also present (yellow arrows). *JET30*: fine mist aerosol formation is absent, unorganized water droplet projectiles are present (yellow arrows). *AIR50*: fine mist aerosol formation is pronounced. *JET50*: pronounced fine mist aerosol formation is visible (white arrows). A single asterisk (^*^) indicates 50 mL/min drillings were illustrated only for comparisons

The coolant spray pattern was observed in the different irrigation modes. In the water jet cooling mode at a flow rate of 16–18 mL/min, the continuous water jet became unstable and showed an intermittent coolant flow (dripping) in the drill’s shaft (JET15). When the chip air was added in AIR15, the coolant flow became continuous (Video 1, Figs. 7 and 8). In JET30, a nice and strong water jet was seen (Video 1, Figs. 7 and 8). Through analyzing the macro-photographs, a pronounced fine mist was observed in groups AIR15 and AIR30 during simulated cavity preparation drilling when there was contact between the drill and the tooth cavity (Figs. 7 and 8) (Table 2). In contrast, fine mist spray components were not observed in the JET15 and JET30 irrigation modes (Video 1, Figs. 7 and 8). The observers’ findings are summarized in Table 2. During simulated drillings with tooth cavity contact, the fine mist was present significantly more often in AIR15 (18/20) than in JET15 (0/20) (*p* < 0.001) and more often in AIR30 (20/0) than in JET30 (0/20) (*p* < 0.001). Single or multiple unorganized water projectile droplets were significantly more frequent in JET15 (14/20) than in AIR15 (1/20) (*p* < 0.001); however, these findings were similar in JET30 (15/20) and AIR30 (19/20) (*p* = 0.182). Cohen’s kappa test showed adequate intra- (0.76 and 0.79) and inter-observer (0.81) reliability in this study.

**Table 2:** The main coolant spray pattern characteristics captured in macro-photographs according to irrigation mode

	Spray pattern^*^			
Coolant mode	Contact with tooth cavity		No contact with tooth cavity	
	Aerosol mist present Yes/no	Isolated water droplets present Yes/no	Aerosol mist present Yes/no	Isolated water droplets present Yes/no
AIR15	18/2	1/19	20/0	0/20
JET15	0/20	14/6	0/20	0/20
AIR30	20/0	19/1	20/0	0/20
JET30	0/20	15/5	0/20	0/20

*AIR15*, spray mist coolant at 15 mL/min; *JET15*, water jet coolant at 15 mL/min; *AIR30*, spray mist coolant at 30 mL/min; *JET30*, water jet coolant at 30 mL/min

^*^Based on image analyses and observers’ decisions

## Discussion

This study confirmed that aerosol reduction is possible when using speed-increasing handpieces in the water jet cooling mode and that pulp temperatures can still be controlled depending on the applied coolant amount. The hypothesis was rejected in terms of intrapulpal heat development and fine mist aerosol formation, while it was kept regarding preparation times.

The current COVID-19 pandemic caused by the coronavirus (SARS-CoV-2) has demonstrated the importance of and concerns regarding existing infection control measures for dental healthcare professionals, as they have an extremely high risk of becoming infected with SARS-CoV-2 [6, 12]. Current preventative protocols recommend patient triage, preoperative mouth rinses, hand hygiene, personal protective equipment, rubber dam isolation, cleaning of contaminated surfaces, and limitation of aerosol-producing procedures [3, 5, 11–13]. Since dental aerosols and droplets can be primary causes of disease transmission, there are some specific recommendations for reducing aerosol spray [16–18]. These include implementing four-hand dentistry using high-volume evacuators with large diameter tips and avoiding the use of high-speed drilling or ultrasonic devices when possible [3, 11].

The duration of time airborne aerosol particles are suspended in the air depends on the weight and size of the droplets [3]. Particles smaller than 5 μm can remain suspended for more than 3 h, while sedimented particles may remain viable for up to 72 h, especially on stainless steel or plastic materials [15, 28]. One method of reducing aerosols and splatter is to use speed-increasing contra-angle handpieces instead of high-speed air-turbines. The water jet mode has recently been introduced as an alternative to block the spray (“chip air”) from dental handpieces. According to the handpiece manufacturers’ measurements and a single recently published investigation, this modification has effectively resulted in a decrease in aerosol leak and fluid droplet dispersion [4]. Our study found that when using water jet irrigation at a coolant flow rate up to 30 mL/min, a fine mist spray pattern was not observed. In contrast, in the AIR15 and AIR30 groups, because of the pre-misted coolant, fine spray mist was clearly visible. These fine mist aerosol airborne particles may remain suspended significantly longer, increasing the risk of infection [4]. Although JET15, similar to JET30, did not cause radial atomization originating from the coolant and drill interaction, from a thermal point of view (mean temperature increase of 6.02 °C with a maximum increase of 6.9 °C), it was clear that JET15 could not be recommended. The accepted thermal threshold for pulp tissue, to prevent pulpal necrosis, is an increase of 5.5 °C [21, 24, 29–32]. Temperature increases of 6 °C or more made heat shock protein 70 immediately detectable [32]. In addition, the reaction of the pulp greatly depends on the general condition of the pulp tissue [24, 30, 33]. The remaining dentin thickness may further influence pulpal coagulation necrosis [32, 34]. The combination of irritating stimuli may lead to clinically symptomless chronic inflammation before progressing to more severe irreversible pulpal damage [30]. Although the JET15 group was the only group with temperature increases higher than the pulp damage threshold, it is important to mention that usual restorative procedures have further thermal “pulp loading” steps. These include the heating effect of light curing units (LCUs) and the effect of the exothermic polymerization reactions of dentin adhesives and resin-composite materials [21]. According to Zarpellon et al., this cumulative thermal load with second and third generation light emitting diode LCUs may be up to 23.2 °C in the pulp chamber [29]. Lynch et al. found that even with 1.39 mm of dentine thickness and only during 10 s of exposure, the mean temperature change can be 13.9 °C [32].

Several in vitro investigations have examined temperature increases in the pulp chambers during tooth preparation. Chua et al. showed that coolant port design (1-port, 3-port, and 4-port) does not significantly influence pulp chamber temperatures using air-turbines [30]. Segal et al. concluded that premium diamond burs may reduce the risk of pulp tissue damage [24]. In the case of medium-grit torpedo diamond drills, premium products produced temperatures that were 5.5 °C lower on average compared to the standard quality drills in air-turbines. Farah showed that the temperature of the cooling fluid can significantly influence pulp chamber temperatures, with a 10 °C coolant temperature causing pulp temperatures that were 23.1 °C lower than the drillings without coolant [19]. In addition, a 23 °C coolant even caused a decrease in pulp temperatures. However, Farah removed the cusps and enamel of tooth crowns to flatten the tooth surface and left only 2.5 mm dentin thickness before test cavities at a maximum of 2 mm were prepared, an important difference from our study. In our opinion, our cavities more reliably simulated a real situation, as they were deeper and narrower. On the other hand, Farah’s drillings lasted a minimum of 60 s (vs. 16–17 s in our study) and a flow rate of 50 mL/min coolant was used, while the drill pressure force was only 0.5 N (vs. 3 N in ours) [19]. The low drilling force applied in that study is certainly among the lowest possible values, even when using air-turbines (0.44–1.83 N); however, air-turbines work with significantly higher revolutions (350,000–450,000 rpm vs. the electric handpiece max. of ~ 200,000 rpm) [2, 23, 26, 35]. Due to these differences in revolution speed and our clinical experiences, a lateral drilling force of 3 N was used in the current experiments. In an earlier study by Watson et al., electric handpieces showed correct performance between 2 and 4.4 N of force [27].

There are two theoretical methods of reducing the atomization of the coolant from the drill’s surface. Reducing revolutions to 60,000–100,000 rpm, according to Sergis et al. [4], may cause a significantly lower radial atomization effect in the water jet cooling mode [4]. However, in that study, the coolant flow rate was not published. Through evaluating the images in Sergis et al.’s results, we assumed that it was > 50 mL/min [4]. Previously, Watson et al. found that drilling at 100,000 rpm caused ~ 10% higher pulp temperatures and longer preparations than drilling at 200,000 rpm. Unfortunately, the exact increase in drilling times was not published [27].

Another potential way to reduce the atomization effect and thus reduce fine misted aerosols is to lower the water coolant flow rate. Based on our study, this seems optimal because cavity preparations may be faster if revolution speeds are not decreased, leading to a shorter exposure. In fact, high-speed handpieces have often been investigated using coolant flow rates of 30 mL/min or more [19, 24, 30]. In a study by Öztürk et al., the low water-cooling group used a 15-mL/min pre-misted coolant, and in some circumstances, even this preparation produced temperature increases above the 5.5 °C threshold [22]. In contrast, in our experiment, the AIR15 method seemed thermally safe, and only the JET15 method resulted in temperature increases similar to the findings of Öztürk et al. (6.02 °C vs. 5.9 °C) [22]. In the current study, increasing the coolant flow rate to 30 mL/min using the water jet irrigation, pulp chamber temperatures did not surpass the pulp tissue damage threshold and fine mist from radial atomization was completely absent.

To effectively control dental cross-infections during this pandemic and even after, the most optimal and safest drilling methods and preparations known should be used. Further studies are needed to investigate other combinations of revolutions and lower water jet coolant flow rates to identify the most ideal combination. However, though in vitro results should be interpreted with caution and further confirmation in real clinical situations is needed, these findings can also be beneficial to increase safety during this crisis.

## Conclusions

Taking into consideration the limitations of this in vitro study, it can be concluded that an effective reduction in fine mist aerosol spray dispersion is possible using the water jet (without spray air) irrigation mode of speed-increasing contra-angle handpieces. In addition, temperature increases in the pulp chambers using water jet irrigation and a coolant flow rate of 30 mL/min was thermally safe, while the efficiency of the preparation remained unaltered. This optimal mode of speed-increasing contra-angle handpieces should be considered for restorative cavity preparations in dental practices.

## Supplementary Information

Below is the link to the electronic supplementary material.

Video 1 Four different coolant modes were examined while drills were positioned in the tooth cavity (MP4 33250 KB)
